# Common Variation at 1q24.1 (*ALDH9A1*) Is a Potential Risk Factor for Renal Cancer

**DOI:** 10.1371/journal.pone.0122589

**Published:** 2015-03-31

**Authors:** Marc Y. R. Henrion, Mark P. Purdue, Ghislaine Scelo, Peter Broderick, Matthew Frampton, Alastair Ritchie, Angela Meade, Peng Li, James McKay, Mattias Johansson, Mark Lathrop, James Larkin, Nathaniel Rothman, Zhaoming Wang, Wong-Ho Chow, Victoria L. Stevens, W. Ryan Diver, Demetrius Albanes, Jarmo Virtamo, Paul Brennan, Timothy Eisen, Stephen Chanock, Richard S. Houlston

**Affiliations:** 1 Division of Genetics and Epidemiology, The Institute of Cancer Research, London, United Kingdom; 2 Division of Cancer Epidemiology and Genetics, Department Health and Human Services, National Cancer Institute, National Institutes of Health, Bethesda, Maryland, United States of America; 3 Division of Cancer Prevention and Population Sciences, Department of Epidemiology, The University of Texas M.D. Anderson Cancer Center, Houston, Texas, United States of America; 4 MRC Clinical Trials Unit at University College London, Aviation House, London, United Kingdom; 5 Royal Marsden NHS Foundation Trust, London, United Kingdom; 6 Cancer Genomics Research Laboratory, Leidos Biomedical Research Inc., Gaithersburg, Maryland, United States of America; 7 Epidemiology Research Program, American Cancer Society, Atlanta, Georgia, United States of America; 8 Department of Chronic Disease Prevention, National Institute for Health and Welfare, Helsinki, Finland; 9 Cambridge University Health Partners, Cambridge, United Kingdom; 10 International Agency for Research on Cancer, Lyon, France; 11 McGill University and Genome Quebec Innovation Centre, Montreal, Quebec, Canada; IFOM, Fondazione Istituto FIRC di Oncologia Molecolare, ITALY

## Abstract

So far six susceptibility loci for renal cell carcinoma (RCC) have been discovered by genome-wide association studies (GWAS). To identify additional RCC common risk loci, we performed a meta-analysis of published GWAS (totalling 2,215 cases and 8,566 controls of Western-European background) with imputation using 1000 Genomes Project and UK10K Project data as reference panels and followed up the most significant association signals [22 single nucleotide polymorphisms (SNPs) and 3 indels in eight genomic regions] in 383 cases and 2,189 controls from The Cancer Genome Atlas (TCGA). A combined analysis identified a promising susceptibility locus mapping to 1q24.1 marked by the imputed SNP rs3845536 (*P*_combined_ =2.30x10^-8^). Specifically, the signal maps to intron 4 of the *ALDH9A1* gene (aldehyde dehydrogenase 9 family, member A1). We further evaluated this potential signal in 2,461 cases and 5,081 controls from the International Agency for Research on Cancer (IARC) GWAS of RCC cases and controls from multiple European regions. In contrast to earlier findings no association was shown in the IARC series (*P*=0.94; *P*_combined_ =2.73x10^-5^). While variation at 1q24.1 represents a potential risk locus for RCC, future replication analyses are required to substantiate our observation.

## Introduction

Worldwide kidney cancer accounts for around 2% of all malignancies the disease affecting 270,000 individuals and causing 116,000 cancer-related deaths each year [1]. In adults 90% of kidney cancers are renal cell carcinomas (RCC) [2].

Besides the well-recognised modifiable risk factors for RCC—smoking and obesity-related traits, as well as the inverse relationship between risk and alcohol consumption, there is strong evidence for an inherited genetic predisposition [3]. Rare germline mutations in *VHL* (von Hippel—Lindau syndrome), *MET* (hereditary papillary renal carcinoma), *BHD* (Birt—Hogg—Dube syndrome) and *FH* (hereditary leiomyomatosis and RCC syndrome) dramatically increase the risk of RCC [4], but contribute little to the overall two-fold familial risk [5]. Evidence for polygenic susceptibility to RCC has recently been vindicated by genome-wide association studies (GWAS) that have identified risk SNPs (single nucleotide polymorphisms) at 2p21, 2q22.3, 8q24.21, 11q13.3, 12p11.33 and 12q24.31 [2,6–9].

To identify additional RCC risk SNPs, we imputed over 10 million SNPs in two published GWAS datasets, using data from the 1000 Genomes Project [10] and UK10K projects as reference (see Materials & Methods for details). This allowed us to recover untyped genotypes, thereby maximising the prospects of identifying novel risk variants for RCC. We then conducted a genome-wide meta-analysis of the two imputed studies.

## Results

For the meta-analysis we made use of data from two previously published GWAS of RCC: (i). UK-GWAS, 1,045 RCC cases genotyped on Illumina Omni Express BeadChips with 2,699 individuals from the Wellcome Trust Case Control Consortium 2 (WTCCC2) 1958 birth cohort and 2,501 UK Blood Service which had been genotyped genotyped on Hap1.2M-Duo arrays serving as controls [2]; (ii) The National Cancer Institute (NCI) GWAS (NCI-GWAS), consisting of four European case-control series, totalling 1,311 cases and 3,424 controls, genotyped on HumanHap HapMap 500, 610 or 660W BeadChips [7].

Post quality control these GWAS provided data on a total of 2,215 cases and 8,566 controls. To maximise identification of novel risk variants, we imputed over 10 million SNPs using 1000 Genomes Project and UK10K data as reference. Quantile-quantile (Q-Q) plots for all SNPs post-imputation did not show substantive over-dispersion (*λ* = 1.02 and 1.01 for UK-GWAS and NCI-GWAS respectively; S1 Fig.).

**Fig 1:**
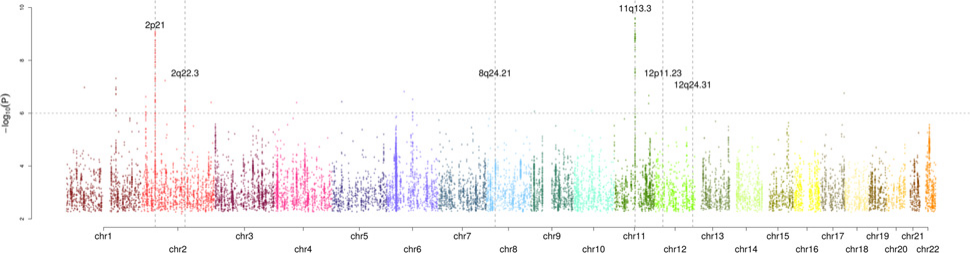
Genome-wide *P*-values (–log_10_ *P*, *y*-axis) plotted against their respective chromosomal positions (*x*-axis). The horizontal line represents the significance threshold level (*P* = 1.0x10^-6^) required for variants to be taken forward to the replication stage. RCC risk loci reported in previous studies are labelled.

We pooled the data from these two GWAS and used an inverse-variance weighted fixed-effects meta analysis model to compute odds ratios (OR), confidence intervals (CI) and *P*-values for each SNP. Results from this meta-analysis, annotated with known risk loci, are shown on Fig. 1. We excluded SNPs that (i) directly mapped to previously published risk loci (2p21, 2q22.3, 8q24.21, 11q13.3, 12p11.33 and 12q24.31; S1 Table), (ii) were in linkage disequilibrium (LD; at a threshold of *r* ^2^ > 0.8) with SNPs from these loci or (iii) had *P*>0.01 in either the UK or the NCI dataset. After applying these filters, we considered 22 SNPs and 3 indels in eight regions of LD that showed evidence for association with RCC risk at *P*<1.0 × 10^-6^ (S2 Table). To validate these potential associations, we conducted replication in cases and controls obtained from combining The Cancer Genome Atlas (TCGA) Kidney Renal Clear Cell Carcinoma (KIRC) and Cancer Genetic Markers of Susceptibility (CGEMS) datasets (383 cases and 2,189 controls; S3 Table).

In an analysis combining these three datasets, rs3845536, mapping to chromosome 1q24.1 (165,650,787 bps; NCBI build 37), achieved genome-wide significance (*P* = 2.30 × 10^-8^; *P*_het_ = 0.24, *I* ^2^ = 29%; Table 1) for association with RCC risk. This association was driven by the NCI (*P* = 9.40x10^-7^) and UK (*P* = 4.61x10^-3^) studies and was not nominally significant in the TCGA study (*P* = 0.16). However, in the latter, smaller, study the effect is of similar size and in the same direction as in the UK and NCI studies, thereby boosting the association signal in the meta-analysis.

**Table 1:** Risk of RCC associated with rs3845536.

						UK						US						TCGA					
locus	nearest genes^a^	variant	position(hg19)	alleles^b^		RAF cases	RAF controls	OR	CI	P_trend_	IS	RAF cases	RAF controls	OR	CI	P_trend_	IS	RAF cases	RAF controls	OR	CI	P_trend_	IS
Iq24.1	*MGST3,ALDH9A1, TMC01,LOC440700*	rs3845536	165,650,787	C	T	0.68	0.64	1.16	(1.05–1.29)	(4.61E–03)	0.99	0.68	0.62	1.30	(1.17–1.44)	9.40E–07	0.99	0.68	0.64	1.14	(0.95–1.37)	1.60E–01	0.83
		rs10918242	165,656,600	A	G	0.67	0.63	1.16	(1.05–1.29)	(3.38E–03)	1.00	0.67	0.61	1.27	(1.15–1.41)	5.28E–06	0.99	0.67	0.64	1.18	(0.99–1.42)	7.05E–02	0.83
		rs34072474	165,656,829	GA	G	0.67	0.63	1.16	(1.05–1.29)	(3.45E–03)	1.00	0.67	0.61	1.27	(1.15–1.41)	4.86E–06	0.99	0.67	0.64	1.18	(0.98–1.41)	7.74E–02	0.83
		rs12036564	165,658,994	A	G	0.67	0.63	1.17	(1.05–1.29)	(2.29E–03)	1.00	0.67	0.61	1.27	(1.15–1.41)	4.93E–06	0.98	0.67	0.63	1.17	(0.98–1.42)	8.18E–02	0.83
		rs7541817	165,659,714	C	T	0.67	0.63	1.16	(1.05–1.28)	(4.47E–03)	1.00	0.67	0.61	1.27	(1.15–1.41)	5.36E–06	0.98	0.67	0.64	1.18	(0.98–1.41)	8.01E–02	0.82
		rs4307543	165,660,029	G	T	0.67	0.63	1.16	(1.05–1.28)	(4.46E–03)	1.00	0.67	0.61	1.27	(1.15–1.41)	5.27E–06	0.98	0.67	0.63	1.17	(0.98–1.40)	8.54E–02	0.82
						OR	CI		P_fixed_	I^2^(%)	P_het_												
meta-analysis^c^ of UK, US, TCGA & IARC study for rs3845536:						0.90	(0.854–0.944)		2.73E–05	82	9.12E–04												

						IARC^d^						UK, US,& TCGA meta-analysis^c^				
locus	nearest genes^a^	variant	position(hg19)	alleles^b^		RAF cases	RAF controls	OR	CI	P_trend_	IS	OR	CI	P_fixed_	I^2^(%)	P_het_
Iq24.1	*MGST3,ALDH9A1, TMC01,LOC440700*	rs3845536	165,650,787	C	T	0.65	0.64	1.00	(0.925–1.075)	9.38E-01	0.99 ^d^	1.21	(1.13–1.30)	2.30E–08	29	0.24
		rs10918242	165,656,600	A	G	n/a	n/a	n/a	n/a	n/a	n/a	1.21	(1.13–1.29)	2.49E–08	0	0.47
		rs34072474	165,656,829	GA	G	n/a	n/a	n/a	n/a	n/a	n/a	1.21	(1.13–1.29)	2.62E–08	0	0.45
		rs12036564	165,658,994	A	G	n/a	n/a	n/a	n/a	n/a	n/a	1.21	(1.13–1.30)	2.36E–08	0	0.46
		rs7541817	165,659,714	C	T	n/a	n/a	n/a	n/a	n/a	n/a	1.21	(1.13–1.29)	3.98E–08	0	0.43
		rs4307543	165,660,029	G	T	n/a	n/a	n/a	n/a	n/a	n/a	1.21	(1.13–1.29)	4.46E–08	0	0.43

Shown are all variants in the locus achieving genome-wide significance (Pfixed<5x10-8) in the combined analysis of UK, NCI and TCGA data. Replication for rs3845536 is also shown.

RAF = risk allele frequency, OR = odds ratio, CI = confidence interval, IS = imputation accuracy score

^a^ nearest genes = genes within 50kb of rs3845536

^b^ alleles are given as risk & other allele

^c^ all meta-analysis results are for an inverse variance weighted, fixed effects model

^d^ the IARC results are for rs3845536 only and are the result of a meta-analysis of 8 studies from various European countries; the IS for each of the 8 studies was 0.99

**Fig 2:**
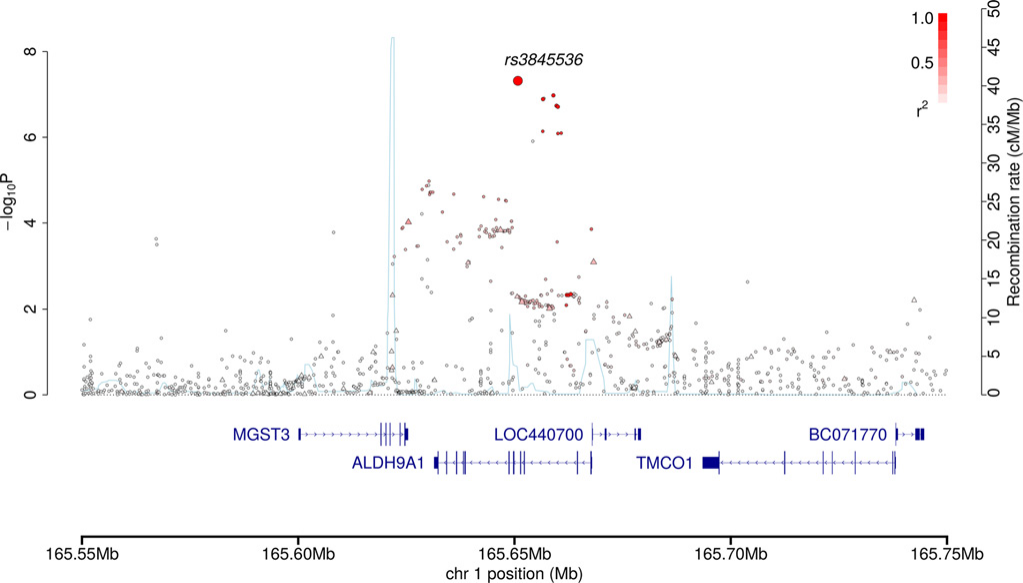
Regional association plot of the 1q24.1 risk locus. The figure shows −log_10_ *P* values (y-axis) versus chromosomal positions (x-axis; NCBI build 37). Genotyped SNPs are shown as triangles, with imputed SNPs as circles. rs3845536 has been highlighted through the use of a larger symbol. Colour intensity is proportional to LD with rs3845536: from white (*r* ^2^ = 0) to red (*r* ^2^ = 1.0). The light blue line indicates genetic recombination rates (estimated from 1000 Genomes Phase 1 CEU data). Nearby genes and transcripts are also shown.

rs3845536 localizes to intron 4 of the *ALDH9A1* gene (aldehyde dehydrogenase, family 9, subfamily a, member 1; MIM 602733; Fig. 2), within a 64kb block of LD. We confirmed the high fidelity of imputation by directly genotyping rs3845536 in a random subset of the UK-GWAS (516 cases, r^2^ = 0.99 and 402 controls, r^2^ = 0.98, Materials and Methods). The RCC risk associated with rs3845536 genotype is compatible with a log-additive model, the OR for risk allele homozygotes being 1.51 (95% CI: 1.29–1.77).

We did not find evidence for interactions between 1q24.1 and any of the previously published risk loci—specifically we evaluated the interaction effects on RCC risk of rs3845536 with SNPs on 2p21 (rs7579899 and rs4953346), 2q22.3 (rs12105918), 8q24.1 (rs6470588 and rs6470589), 11q13.3 (rs7105934), 12p11.23 (rs718314) and 12q24.31 (rs4765623). The assumption of independent RCC risk loci was supported by the lack of significant interaction terms between the risk loci (*i*.*e*. *P* > 0.05; S4 Table).

Using publicly available mRNA expression data, we evaluated the potential for *cis*-regulation of *ALDH9A1* or other nearby gene by rs3845536 variation. There was no statistically significant relationship between the genotype of rs3845536 or a SNP in LD with rs3845536 (at r^2^>0.8) and expression of *ALDH9A1* and the nearby transcripts *MGST3* and *TMCO1* (expression data for transcripts LOC440700 and BC071770, also in the region, were not available). Further, a Haploreg and RegulomeDb search did not yield evidence for rs3845536 or a correlated SNP to locate within a transcription regulatory region (data not shown). We also made use of TCGA clear cell data to examine the frequency of mutation of *ALDH9A1*, *MGST3*, *LOC440700* and *TMCO1* in renal cancer [11]. None of these genes have mutational frequencies in RCC >1% (no data were available for transcript BC071770).

To further examine this association we made use of data from the International Agency for Research on Cancer (IARC) GWAS of RCC which was based on eight independent case-control series from different European countries with 41.4% of cases from Western and Northern Europe, and 58.6% from Central and Eastern Europe. In the IARC series there was no evidence for an association between rs3845536 and risk of RCC (*P* = 0.94; Table 1). Hence overall, the association strength was markedly reduced with concomitant significant heterogeneity with inclusion of the IARC dataset *(P* = 2.73 x 10^-5^, *P*_het_ = 9.1 x 10^-4^, I^2^ = 82%; Table 1).

## Discussion

We report a newly identified common variant on chromosome 1q24.1 annotating a potential RCC susceptibility locus candidate. If confirmed by additional studies there is a high likelihood that the functional basis of the 1q24.1 risk locus is mediated through *ALDH9A1 a priori* since the region of association is small and rs3845536 is intronic to *ALDH9A1*. Although we did not observe an association between rs3845536 genotype and *ALDH9A1* expression, a subtle relationship between the two, such as a cumulative, long term interaction, remains a possibility.

The ALDH gene superfamily is documented [12] to include a variety of isozymes involved in the metabolism of aldehydes generated from chemically diverse endogenous and exogenous precursors. Aldehyde-mediated effects vary from homeostatic and therapeutic to cytotoxic, and genotoxic and several ALDHs have been implicated in human disease phenotypes or pathophysiologies [12]. *ALDH9A1* encodes γ-trimethylaminobutyraldehyde dehydrogenase that participates in the metabolism of γ-aminobutyraldehyde and aminoaldehydes derived from polyamines [12]. High levels of *ALDH9A1* expression are seen in the kidney [13] with significant enrichment of dehydrogenases including *ALDH9A1* in RCC [14]. TNF signalling is well established to play a role in RCC development [15] and it is notable that *ALDH9A1* influences expression of TNF alpha induced protein 3 [16]. Although speculative these data are consistent with the hypothesis of xenobiotic metabolism associated with apoptosis and tumorigenesis playing a role in RCC oncogenesis. While our finding adds evidence that *ALDH9A1* is implicated in RCC development, further studies are required to determine the variants that are functionally relevant.

To interrogate whether rs3845536 has pleiotropic effects on the risks of other cancer types, we investigated the association with colorectal [17] and lung cancers [18], acute lymphoblastic leukaemia [19], multiple myeloma [20], glioma [21] and meningioma [22] using data from previously reported GWAS. However, our data did not support this hypothesis and we did not observe, for any of these cancers, a significant effect of rs3845536 genotype (or a correlated SNP at r^2^≥0.8) on tumor risk.

In summary, we report a potential RCC risk susceptibility locus candidate at rs3845536. This finding implicates genetic variation in *ALDH9A1* in the development of RCC. Similar to other GWAS hits, rs3845536 is a common variant and confers moderate risk of RCC. However compelling our finding is from analysis of UK, NCI and TCGA data due to the failure to validate the association in the IARC series the observation has to be viewed with a degree of caution at this juncture and further replication is required. We note that due to both the modest size of our discovery dataset and the fact that published RCC susceptibility loci at 2p21, 2q22.3, 8q24.21, 11q13.3, 12p11.33 and 12q24.31 account for <5% of the familial risk additional risk variants are likely to be identifiable through expanded GWAS analyses.

## Materials and Methods

### Ethics statement

Collection of blood samples and clinico-pathological information from all subjects was undertaken with written informed consent with ethical board approvals from the Royal Marsden NHS Hospitals Trust (CCR 1552/1922) and the United Kingdom Multicentre Research Ethics Board (07/MRE01/10). Details about Ethics approval for the NCI, TCGA and IARC studies are detailed previously [7].

### Subjects and datasets

GWAS datasets have been previously reported [2]. (i) UK-GWAS was based on 1,045 RCC cases (including 590 clear cell carcinomas (CCCs), 42 papillary carcinomas (PCs), 33 chromophobe carcinomas (CCs) and 19 mixed or other histological subtypes) genotyped using Human OmniExpress-12 BeadChips, with 856 cases from the MRC SORCE trial and 189 cases collected through The Institute of Cancer Research (ICR) and Royal Marsden NHS Hospitals Trust and 5,200 controls genotyped using Hap1.2M-Duo Custom array with 2,699 individuals from the Wellcome Trust Case Control Consortium 2 (WTCCC2) 1958 birth cohort and 2,501 from the UK Blood Service. (ii) NCI-GWAS was based on 1,453 RCC cases and 3,599 controls of European background genotyped using Illumina HumanHap HapMap 500, 610 or 660W BeadChips. Data were publicly available on 1,311 cases (including 534 CCCs, 93 PCs, 86 other histological subtypes) and 3,424 controls [7].

As we previously described [2], we applied a number of pre-specified quality control metrics to the data. Specifically we used the following criteria to exclude individuals: overall successfully genotyped SNPs < 97%, discordant sex, outliers in a plot of heterozygosity versus missingness, duplication or relatedness to the estimated identity by descent (IBD) > 0.185 or evidence of non- European ancestry by PCA-based analysis using HapMap reference samples (S2 Fig.). SNP exclusion criteria included: call rate <95%; different missing genotype rates between cases and controls at *P* < 10^-5^; MAF < 0.01; departure from Hardy—Weinberg equilibrium in controls at *P* < 10^-5^. An overview of all sample exclusions is given in S3 Fig. Adequacy of the case—control matching was assessed by inspection of Q—Q plots of test statistics and by means of the inflation factor λ_GC_.

### Replication series

For replication, we used, as detailed previously [2], data from TCGA and IARC. Briefly, the TCGA RCC clear cell cases (KIRC study, accession number phs000178.v7.p6) were genotyped using the Affymetrix Genome-Wide Human SNP Array 6.0. For controls we made use of data on healthy individuals from the CGEMS breast and prostate cancer study, genotyped using Illumina HumanHap550 and Phase 1A HumanHap300+Phase 1BHumanHap240 Beadchips respectively. Both cases and controls were formally examined for an overlap with the NCI GWAS samples. Any TCGA or CGEMS sample found to be a duplicate of or related to a sample from the NCI GWAS was removed from the replication dataset. After further checking for relatedness and European ancestry 383 cases and 2,189 controls constituted the TCGA/CGEMS replication series. The International Agency for Research on Cancer (IARC) GWAS consisted of 2,461 RCC cases (including 1,340 CCCs, 95 PCs, 88 other histological subtypes) and 5,081 controls of European background from eight European studies) and has previously been described [7]. Genotyping of cases and controls was performed using either Illumina HumanHap300, 550 or 610 Quad Beadchips. Data derived from the three arrays were imputed to recover rs3845536 genotype.

### Statistical and bioinformatic analyses

R (v3.02) and SNPTEST (v2.4.1) software were used for analysis. Association between individual SNPs and RCC risk was evaluated by the Cochran—Armitage trend test. Unconditional logistic regression was used to calculate ORs and associated 95% CIs. The UK-GWAS did not require any covariates to adjust for, the NCI-GWAS required adjusting for study centre and the TCGA-GWAS required adjusting for the first principal component. Phasing of GWAS SNP genotypes was performed using SHAPEIT v2.644. Untyped SNPs were imputed using IMPUTEv2 (v2.3.0) with data from the 1000 Genomes Project (Phase 1 integrated variant set, v3.20101123, released on the IMPUTEv2 website on 9 December 2013) and UK10K (ALSPAC, EGAS00001000090 / EGAD00001000195, and TwinsUK, EGAS00001000108 / EGAD00001000194, studies only) used as reference panels. Analysis of imputed data was conducted using SNPTEST v2.4.1 to account for uncertainties in SNP prediction. Association meta-analyses only included markers with info scores >0.4, imputed call rates/SNP >0.9 (UK & NCI studies) and MAFs >0.005. Meta-analyses were carried out with the R package meta v2.4–1, using the genotype probabilities from IMPUTEv2 for untyped SNPs. Heterogeneity was assessed using Cochran's *Q* statistic and the proportion of the total variation due to heterogeneity was assessed using the *I* ^2^ statistic.

HapMap recombination rate (cM/Mb) was used to define LD blocks. The recombination rate defined using the Oxford recombination hotspots and on the basis of the distribution of CIs defined by Gabriel and co-workers [23].

The fidelity of imputation, as assessed by the concordance between imputed and directly genotyped SNPs, was examined in a random subset of samples from the UK-GWAS. To quantify the fidelity of imputation we calculated Pearson’s correlation coefficient r^2^ between directly genotyped values (counting the number of reference alleles, taking discrete values in {0, 1, 2}) and the imputed genotypes (taking real values in the interval [0,2]).

The familial relative risk of RCC attributable to a specific variant was calculated using the formula from [24]:

$$\lambda^{*}\;=\;\frac{p{\left(pr_{2}+qr_{1}\right)}^{2}+q{\left(pr_{1}+q\right)}^{2}}{{\left(p^{2}r_{2}+2pqr_{1}+q^{2}\right)}^{2}},$$

where the overall sibling relative risk *λ*_0_ for RCC is 2.45 [5].

Fig. 2 has been produced using visPIG [25].

### Analysis of TCGA data

The associations of SNP genotype with gene expression in RCC was investigated using TCGA data generated using Agilent 244K Custom G4502A arrays. The frequency of mutations was obtained using the CBioPortal for Cancer Genomics web server.

### Supporting Information

Supporting information is available at *PLOS ONE* online.

### URLs

R Core Team (2013). R: A language and environment for statistical computing. R Foundation for Statistical Computing, Vienna, Austria. URL http://www.R-project.org/.

Illumina: http://www.illumina.com

dbSNP: http://www.ncbi.nlm.nih.gov/projects/SNP

HapMap: http://www.hapmap.org

1000Genomes: http://www.1000genomes.org

visPIG: http://vispig.icr.ac.uk

IMPUTE: https://mathgen.stats.ox.ac.uk/impute/impute

SNPTEST: http://www.stats.ox.ac.uk/~marchini/software/gwas/snptest

cBioPortal for Cancer Genomics: http://www.cbioportal.org

Wellcome Trust Case Control Consortium: www.wtccc.org.uk

Mendelian Inheritance In Man: http://www.ncbi.nlm.nih.gov/omim

The Cancer Genome Atlas project: http://cancergenome.nih.gov

Genevar (GENe Expression VARiation): http://www.sanger.ac.uk/resources

SORCE: http://www.ctu.mrc.ac.uk

Cancer Genetic Markers of Susceptibility (*CGEMS*): cgems.cancer.gov

## Supporting Information

S1 Dataset UK & NCI association test results with meta-analysis results. Tab-delimited ASCII text file with one header row.
(TXT)

S1 Fig Q-Q plots of Cochran-Armitage trend test statistics for association based on meta-analysis of UK-GWAS and NCI-GWAS pre-imputation (a-b); post-imputation (e-h) and rare SNPs post-imputation (i-l). The identity line is indicated as a blue dashed line.
(TIF)

S2 Fig first two principal components of the UK and NCI datasets, as used for removing samples based on ancestry during quality control. Case and control samples are indicated as grey and black crosses, with the HapMap reference populations shown as bold coloured discs.
(TIF)

S3 Fig GWAS data quality control. Details are provided of samples, SNPs and quality control (QC) used in each GWAS.
(TIF)

S1 Table Evidence for association at previously reported RCC susceptibility loci. At each locus values are given for the previously reported SNPs and the lead SNP in this study.
(PDF)

S2 Table UK & NCI meta-analysis for all variants taken through to the replication stage. (PDF)

S3 Table UK, NCI & TCGA meta-analysis for all variants taken through to the replication stage. Shown in bold are the variants achieving *P*_fixed_<5x10^-8^.
(PDF)

S4 Table significance of the interaction terms of rs3845536 with previously published risk SNPs for RCC. (PDF)
