# Supplementary figures and images for: Common Variation at 1q24.1 (ALDH9A1) Is a Potential Risk Factor for Renal Cancer
Source: PLoS One. 2015 Mar 31;10(3):e0122589. doi: 10.1371/journal.pone.0122589 (PMC4380462; doi:10.1371/journal.pone.0122589)

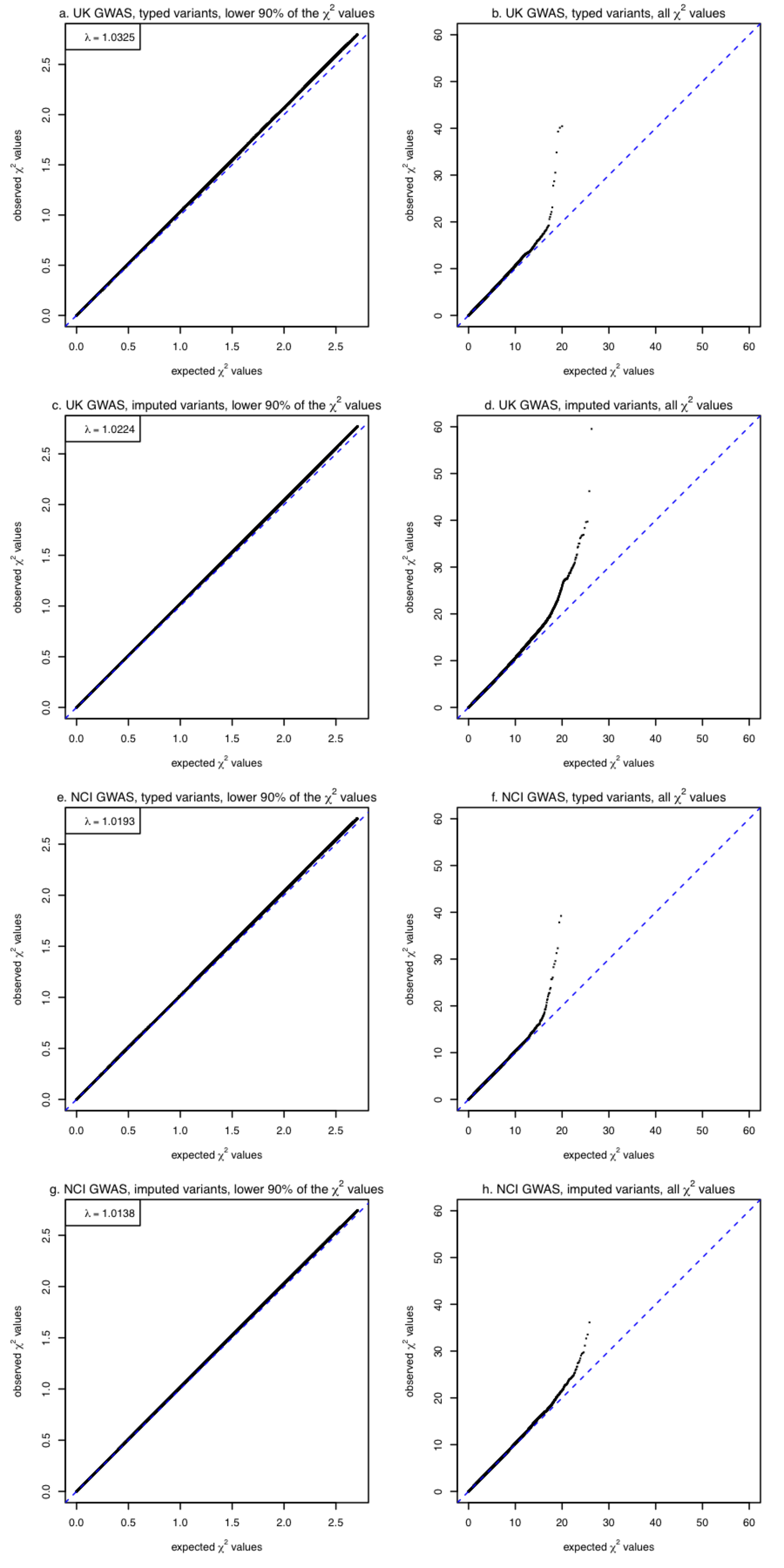

Supplement: S1 Fig — The identity line is indicated as a blue dashed line. (TIF) [file pone.0122589.s002.tif]

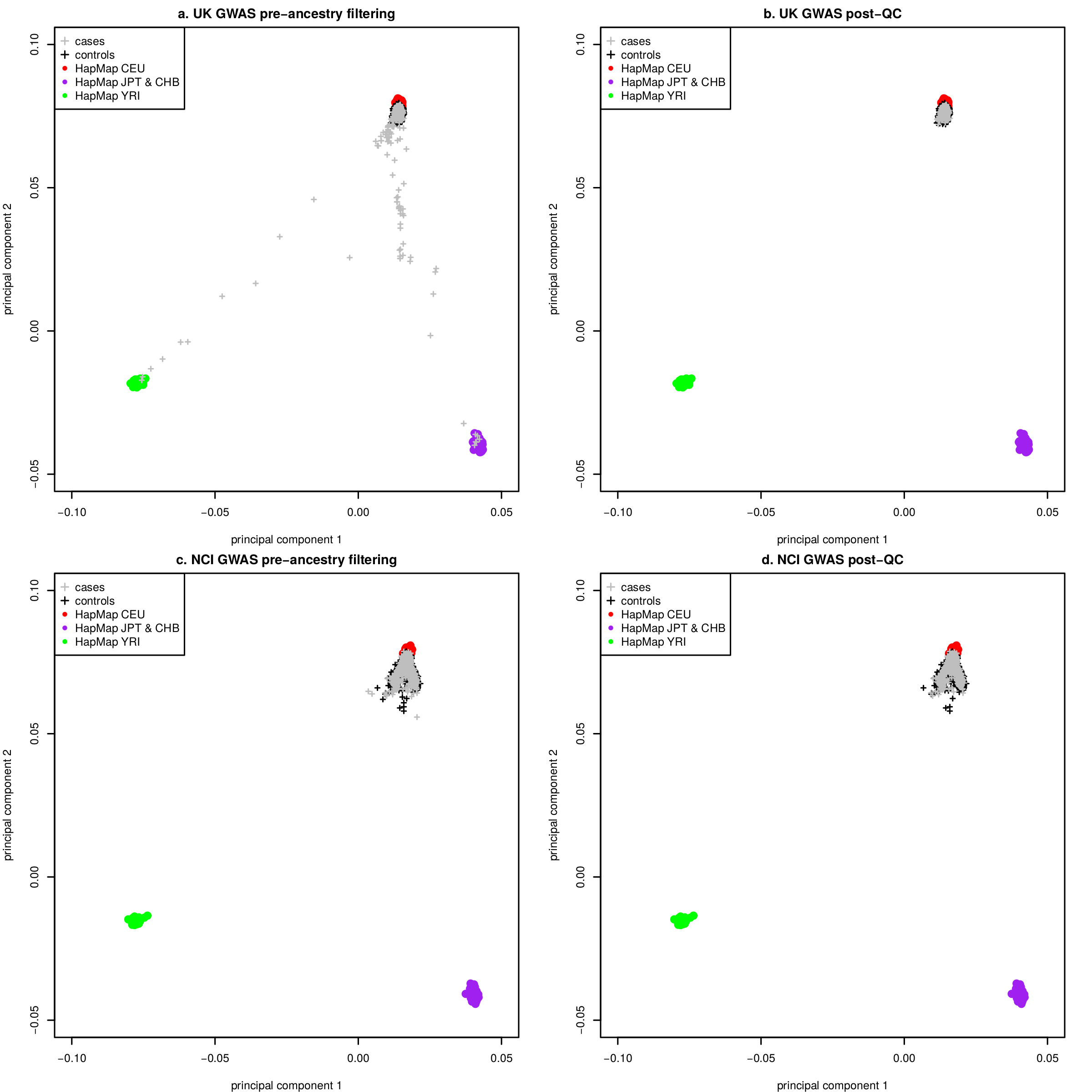

Supplement: S2 Fig — Case and control samples are indicated as grey and black crosses, with the HapMap reference populations shown as bold coloured discs. (TIF) [file pone.0122589.s003.tif]

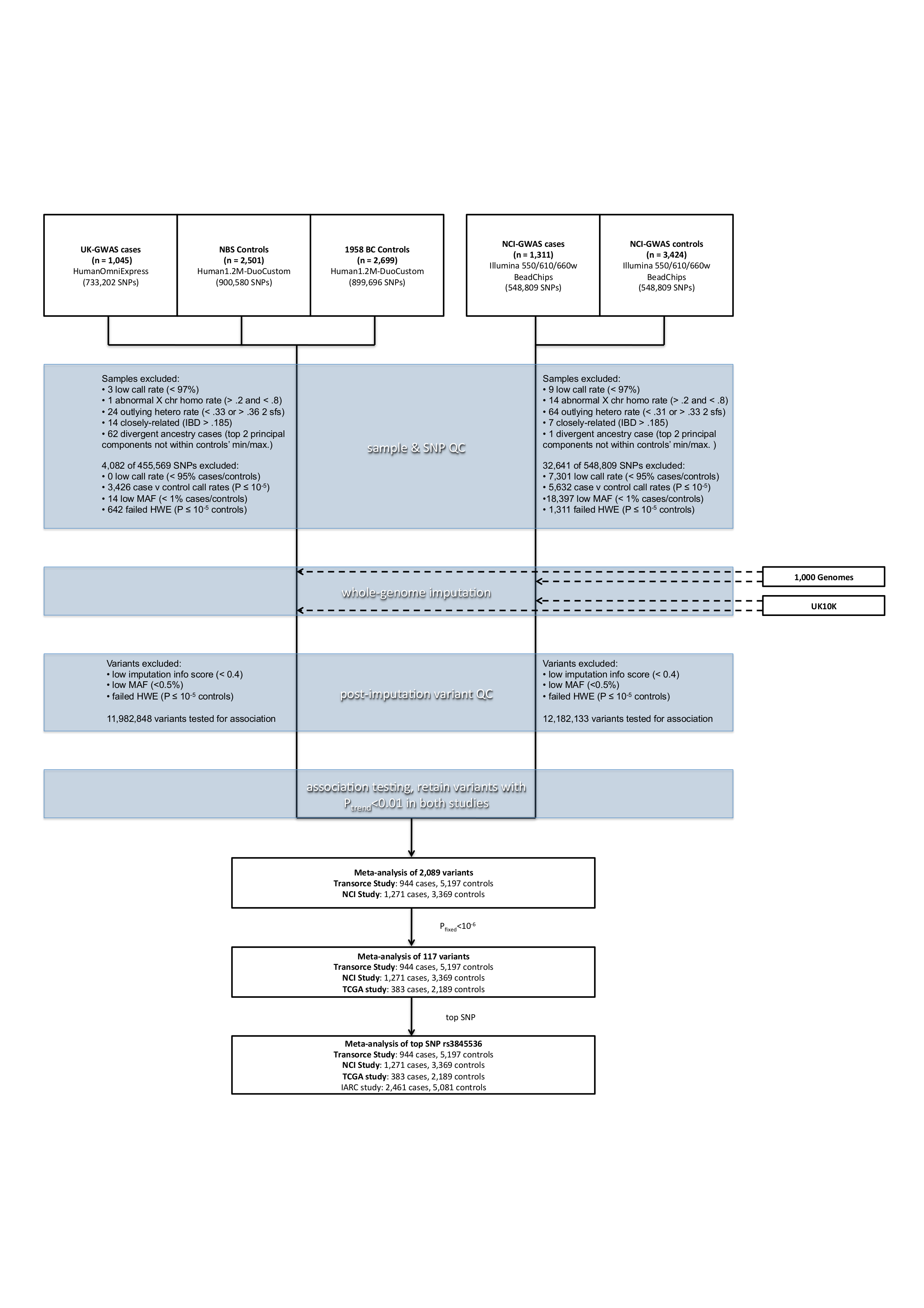

Supplement: S3 Fig — Details are provided of samples, SNPs and quality control (QC) used in each GWAS. (TIF) [file pone.0122589.s004.tif]
