# Supplementary material for: Common Variation at 1q24.1 (ALDH9A1) Is a Potential Risk Factor for Renal Cancer
Source: PLoS One. 2015 Mar 31;10(3):e0122589. doi: 10.1371/journal.pone.0122589 (PMC4380462; doi:10.1371/journal.pone.0122589)
Supplement: S1 Table — At each locus values are given for the previously reported SNPs and the lead SNP in this study. (PDF) [file pone.0122589.s005.pdf]

Supplementary Table S1: Evidence for association at previously reported RCC susceptibility loci. At each locus values are given for the previously reported SNPs and the lead SNP in this study.

								individual datasets								meta-analysis					
								UK				NCI				fixed effects			random effects		
locus	nearest gene(s)	published variant	lead variant in harmonised data[a]	position (hg19)	alleles[b]		MAF[c]	OR	SE	P_{trend}	IS[d]	OR	SE	P_{trend}	IS[d]	OR	SE	P	OR	SE	P
2p21	EPAS1	rs7579899		46,537,604	A	G	0.39	1.12	0.05	2.58E-02	0.99	1.18	0.05	1.68E-03	DT	1.15	0.04	1.43E-04	1.15	0.04	1.43E-04
2p21	EPAS1	rs11894252		46,533,376	T	C	0.40	1.11	0.05	3.56E-02	0.99	1.19	0.05	8.78E-04	DT	1.15	0.04	1.23E-04	1.15	0.04	1.23E-04
2p21	EPAS1	rs9679290		46,557,644	C	G	0.46	1.16	0.05	2.71E-03	1.00	1.30	0.05	1.48E-07	1.00	1.23	0.04	4.71E-09	1.23	0.06	1.98E-04
2p21	EPAS1	rs4953346		46,558,208	G	T	0.46	1.16	0.05	2.83E-03	1.00	1.30	0.05	1.55E-07	1.00	1.23	0.04	5.24E-09	1.23	0.06	2.20E-04
2p21	EPAS1	rs12617313		46,559,776	T	A	0.47	1.14	0.05	8.41E-03	0.97	1.31	0.05	5.82E-08	0.99	1.23	0.04	1.13E-08	1.23	0.07	3.60E-03
2p21	EPAS1		rs4953345	46,552,601	A	T	0.50	1.21	0.05	1.60E-04	0.95	1.28	0.05	9.66E-07	0.99	1.25	0.04	5.26E-10	1.25	0.04	5.26E-10
2q22.3	ZEB2	rs12105918		145,208,193	C	T	0.06	1.50	0.11	1.41E-04	DT	1.39	0.10	1.30E-03	DT	1.44	0.07	3.38E-07	1.44	0.07	3.38E-07
2q22.3	ZEB2	rs13389578		145,216,048	C	T	0.08	1.32	0.09	2.18E-03	DT	1.37	0.09	3.23E-04	DT	1.34	0.06	1.79E-06	1.34	0.06	1.79E-06
2q22.3	ZEB2		rs72858496	145,209,916	T	C	0.06	1.52	0.11	8.78E-05	0.99	1.40	0.10	9.82E-04	0.99	1.46	0.07	1.72E-07	1.46	0.07	1.72E-07
8q24.1	MYC,PVT1	rs35252396			CG	AC		two base-pair substitution variant not included on UK & NCI arrays and not included in the imputation reference panels													
8q24.1	MYC,PVT1	rs6470588[e]		128,889,371	C	A	0.24	1.09	0.07	2.07E-01	0.77	1.14	0.05	1.26E-02	DT	1.11	0.04	3.26E-03	1.11	0.04	3.26E-03
8q24.1	MYC,PVT1	rs6470589[e]		128,889,372	G	C	0.24	1.09	0.07	1.83E-01	0.77	1.14	0.05	1.21E-02	0.98	1.12	0.04	2.72E-03	1.12	0.04	2.72E-03
8q24.1	MYC,PVT1		8-128966831	128,966,831	A	G	0.01	3.61	0.37	5.51E-04	0.73	1.75	0.34	9.84E-02	0.71	2.51	0.24	1.15E-04	2.51	0.36	1.11E-02
11q13.3	AX746803,AK094674	rs7105934		69,239,741	G	A	0.08	1.62	0.09	2.34E-07	0.99	1.38	0.10	8.67E-04	DT	1.50	0.07	4.93E-09	1.50	0.08	2.83E-07
11q13.3	AX746803,AK094674		rs11263654	69,238,123	C	T	0.08	1.66	0.09	6.06E-08	0.99	1.41	0.10	5.44E-04	1.00	1.53	0.07	1.33E-09	1.53	0.08	2.35E-07
12p11.23	SSPN,ITPR2	rs718314		26,453,283	G	A	0.24	1.18	0.06	3.87E-03	DT	1.06	0.06	2.82E-01	DT	1.12	0.04	4.17E-03	1.12	0.05	3.31E-02
12p11.23	SSPN,ITPR2	rs1049380		26,489,544	G	T	0.27	1.04	0.06	4.99E-01	DT	1.06	0.05	3.21E-01	DT	1.05	0.04	2.32E-01	1.05	0.04	2.32E-01
12p11.23	SSPN,ITPR2		rs187598091	26,188,405	A	T	0.01	4.96	0.53	2.49E-03	0.53	1.04	0.25	8.88E-01	0.78	2.19	0.18	1.05E-05	2.19	0.82	3.39E-01
12q24.31	SCARB1,JB074994	rs4765623		125,320,850	T	C	0.34	1.20	0.05	5.84E-04	0.99	1.10	0.05	7.49E-02	DT	1.15	0.04	2.30E-04	1.15	0.04	1.55E-03
12q24.31	SCARB1,JB074994		rs12825160	125,224,347	A	G	0.01	1.88	0.32	5.21E-02	0.71	2.03	0.23	2.27E-03	0.83	1.95	0.16	4.43E-05	1.95	0.16	4.43E-05

MAF = minor allele frequency, OR = odds ratio, SE = standard error of the log odds ration, IS = imputation accuracy score (info score from IMPUTEv2), DT = directly typed

[a] lead variant = variant within 500kb of the topmost published variant with lowest fixed effects meta P-value

[b] alleles are given as risk & other allele

[c] minor allele frequency is from UK controls

[d] imputation quality score is provided where applicable (scale: 0-1, variants with a score <0.4 were discarded)

[e] the two base-pair subsitution rs35252396 is often annotated as these two consecutive SNPs
