# Supplementary material for: Common Variation at 1q24.1 (ALDH9A1) Is a Potential Risk Factor for Renal Cancer
Source: PLoS One. 2015 Mar 31;10(3):e0122589. doi: 10.1371/journal.pone.0122589 (PMC4380462; doi:10.1371/journal.pone.0122589)
Supplement: S2 Table — (PDF) [file pone.0122589.s006.pdf]

Supplementary Table S2: UK & NCI meta-analysis for all variants taken through to the replication stage.

						UK						NCI						meta analysis[c]			
locus	nearest gene(s)[a]	variant	position (hg19)	alleles[b]		RAF cases	RAF controls	OR	CI	P_{trend}	IS	RAF cases	RAF controls	OR	CI	P_{trend}	IS	OR	P_{fixed}	I^2 (%)	P_{het}
1q24.1	*MGST3, ALDH9A1, TMC01, LOC440700*	rs3845536	165,650,787	C	T	0.68	0.64	1.16	(1.05- 1.29)	4.61E-03	0.99	0.68	0.62	1.30	(1.17-1.44)	9.40E-07	0.99	1.23	4.81E-08	56	0.13
		rs11583089	165,656,537	A	T	0.72	0.68	1.19	(1.08- 1.33)	9.10E-04	1.00	0.71	0.67	1.22	(1.10-1.36)	2.23E-04	0.99	1.21	7.26E-07	0	0.77
		rs10918242	165,656,600	A	G	0.67	0.63	1.16	(1.05- 1.29)	3.38E-03	1.00	0.67	0.61	1.27	(1.15-1.41)	5.28E-06	0.99	1.22	1.29E-07	31	0.23
		rs34072474	165,656,829	GA	G	0.67	0.63	1.16	(1.05- 1.29)	3.45E-03	1.00	0.67	0.61	1.27	(1.15-1.41)	4.86E-06	0.99	1.22	1.24E-07	33	0.22
		rs12036561	165,658,994	A	G	0.67	0.63	1.17	(1.05- 1.29)	2.99E-03	1.00	0.67	0.61	1.27	(1.15-1.41)	4.93E-06	0.98	1.22	1.06E-07	29	0.24
		rs7541817	165,659,714	C	T	0.67	0.63	1.16	(1.05- 1.28)	4.47E-03	1.00	0.67	0.61	1.27	(1.15-1.41)	5.36E-06	0.98	1.21	1.84E-07	37	0.21
		rs4307543	165,660,029	G	T	0.67	0.63	1.16	(1.05- 1.28)	4.46E-03	1.00	0.67	0.61	1.27	(1.15-1.41)	5.72E-06	0.98	1.21	1.94E-07	36	0.21
		rs7542184	165,660,041	C	A	0.72	0.68	1.19	(1.07- 1.32)	1.19E-03	1.00	0.71	0.67	1.23	(1.10-1.36)	1.86E-04	0.98	1.21	8.17E-07	0	0.70
		rs72698083	165,660,794	G	C	0.72	0.68	1.19	(1.07- 1.32)	1.17E-03	1.00	0.71	0.67	1.23	(1.10-1.36)	1.85E-04	0.98	1.21	8.00E-07	0	0.70
2p24.3		rs1560445	15,237,682	G	A	0.73	0.68	1.24	(1.11- 1.38)	1.00E-04	0.95	0.73	0.71	1.18	(1.06-1.32)	2.34E-03	DT	1.21	9.28E-07	0	0.57
		rs1835468	15,239,211	G	A	0.73	0.68	1.24	(1.11- 1.38)	9.40E-05	0.96	0.73	0.71	1.19	(1.07-1.33)	1.56E-03	1.00	1.22	5.65E-07	0	0.63
		rs13002707	15,241,629	T	A	0.73	0.69	1.23	(1.11- 1.37)	1.15E-04	0.97	0.73	0.71	1.19	(1.06-1.32)	2.08E-03	1.00	1.21	9.21E-07	0	0.61
		rs7573899	15,241,637	T	C	0.73	0.69	1.23	(1.11- 1.37)	1.21E-04	0.97	0.73	0.71	1.19	(1.07-1.33)	1.96E-03	1.00	1.21	9.05E-07	0	0.63
		rs10196061	15,244,929	C	T	0.73	0.69	1.23	(1.11- 1.37)	1.37E-04	0.97	0.73	0.71	1.19	(1.07-1.33)	1.80E-03	1.00	1.21	9.16E-07	0	0.66
		rs13385654	15,252,659	T	C	0.72	0.68	1.25	(1.12- 1.39)	4.28E-05	0.97	0.71	0.69	1.19	(1.07-1.33)	1.34E-03	0.99	1.22	2.36E-07	0	0.57
		rs11695054	15,254,827	G	A	0.73	0.69	1.23	(1.11- 1.37)	1.29E-04	0.99	0.73	0.71	1.19	(1.07-1.33)	1.92E-03	1.00	1.21	9.18E-07	0	0.66
2p12	*REG3G*	2-79243058	79,243,058	AACAG	A	0.02	0.01	3.87	(1.99- 7.53)	6.53E-05	0.67	0.01	0.01	4.41	(2.00-9.71)	2.33E-04	0.57	4.09	5.81E-08	0	0.81
2q37.1	*ITM2C, GPR55, LOC151484*	rs181676155	231,787,699	A	G	0.01	0.01	7.46	(2.92-19.09)	2.75E-05	0.59	0.01	0.01	3.05	(1.55-6.00)	1.22E-03	0.71	4.14	3.88E-07	56	0.13
5p13.3	*PDZD2*	rs10054504	32,000,483	T	C	0.14	0.11	1.41	(1.21- 1.65)	1.29E-05	DT	0.14	0.12	1.24	(1.07-1.44)	4.11E-03	DT	1.32	3.68E-07	28	0.24
6q14.3		rs78793258	85,749,252	G	A	0.09	0.06	1.43	(1.17- 1.73)	3.61E-04	0.98	0.10	0.08	1.40	(1.17-1.68)	2.31E-04	0.98	1.41	2.98E-07	0	0.89
		rs74439948	85,766,266	G	A	0.09	0.06	1.41	(1.16- 1.72)	4.60E-04	0.99	0.10	0.08	1.37	(1.14-1.63)	5.95E-04	0.99	1.39	9.66E-07	0	0.80
		rs7765284	85,777,822	A	C	0.09	0.06	1.42	(1.17- 1.72)	3.73E-04	DT	0.10	0.08	1.36	(1.14-1.62)	6.86E-04	DT	1.39	9.22E-07	0	0.75
10q21.2	*ANK3*	rs149748210	62,066,205	A	ATATATATC	0.47	0.43	1.26	(1.12- 1.41)	1.05E-04	0.73	0.49	0.49	1.20	(1.07-1.34)	1.93E-03	0.76	1.23	8.12E-07	0	0.57
11q23.3		rs113878013	114,385,626	C	T	0.01	0.00	4.67	(2.13-10.22)	1.14E-04	0.78	0.01	0.01	2.98	(1.58-5.60)	7.25E-04	0.78	3.56	4.34E-07	0	0.38
		rs7942540	114,815,288	A	G	0.02	0.01	5.13	(2.41-10.91)	2.24E-05	0.48	0.02	0.01	2.89	(1.51-5.53)	1.42E-03	0.60	3.69	2.16E-07	21	0.26

RAF = risk allele frequency, OR = odds ratio, CI = confidence interval, IS = imputation accuracy score (info score from IMPUTEv2), DT = directly typed

[a] nearest gene(s) = genes within 50kb of the variant with the lowest meta analysis P

[b] alleles are given as risk & other allele

[c] all meta analysis results are for an inverse variance weighted, fixed effect model
