# Supplementary material for: Common Variation at 1q24.1 (ALDH9A1) Is a Potential Risk Factor for Renal Cancer
Source: PLoS One. 2015 Mar 31;10(3):e0122589. doi: 10.1371/journal.pone.0122589 (PMC4380462; doi:10.1371/journal.pone.0122589)
Supplement: S3 Table — Shown in bold are the variants achieving P fixed<5x10^-8^. (PDF) [file pone.0122589.s007.pdf]

Supplementary Table S3: UK, NCI & TCGA meta-analysis for all variants taken through to the replication stage. Shown in bold are the variants achieving $P_{fixed}<5x10^{-8}$.

						UK						NCI						TCGA						meta analysis					
locus	nearest gene(s)[a]	variant	position (hg19)	alleles[b]		cases genotype counts[c]	controls genotype counts[c]	OR	CI	P	IS	cases genotype counts[c]	controls genotype counts[c]	OR	CI	P	IS	cases genotype counts[c]	controls genotype counts[c]	OR	CI	P	IS	OR_{fixed}	CI_{fixed}	P_{fixed}	P_{random}	I^2 (%)	P_{het}
		**rs3845536**	**165,650,787**	**C**	**T**	**446.55 / 388.01 / 109.44**	**2167.6 / 2365.6 / 663.81**	**1.16**	**(1.05- 1.29)**	**4.61E-03**	**0.99**	**578.9 / 572.31 / 119.79**	**1284.1 / 1600.2 / 484.71**	**1.30**	**(1.17-1.44)**	**9.40E-07**	**0.99**	**171.42 / 174.39 / 37.141**	**916.14 / 988.98 / 283.7**	**1.14**	**(0.95-1.37)**	**1.60E-01**	**0.83**	**1.21**	**(1.13-1.30)**	**2.30E-08**	**7.22E-06**	**29**	**0.24**
		rs11583089	165,656,537	A	T	502.1 / 355.21 / 86.69	2416.5 / 2248.8 / 531.64	1.19	(1.08- 1.33)	9.10E-04	1.00	640.69 / 524.43 / 105.88	1490.9 / 1502.1 / 376.02	1.22	(1.10-1.36)	2.23E-04	0.99	194.16 / 160.58 / 28.229	1025.8 / 936.12 / 226.89	1.19	(0.98-1.43)	7.45E-02	0.82	1.21	(1.12-1.29)	1.42E-07	1.42E-07	0	0.94
		**rs10918242**	**165,656,600**	**A**	**G**	**428.08 / 403.84 / 112.09**	**2084.8 / 2398.3 / 713.85**	**1.16**	**(1.05- 1.29)**	**3.38E-03**	**1.00**	**554.3 / 583.18 / 133.51**	**1231.5 / 1615.9 / 521.61**	**1.27**	**(1.15-1.41)**	**5.28E-06**	**0.99**	**170.67 / 174.27 / 38.035**	**888.53 / 1003.5 / 296.82**	**1.18**	**(0.99-1.42)**	**7.05E-02**	**0.83**	**1.21**	**(1.13-1.29)**	**2.49E-08**	**2.49E-08**	**0**	**0.47**
		**rs34072474**	**165,656,829**	**GA**	**G**	**428.06 / 403.86 / 112.07**	**2085.8 / 2397.2 / 714.03**	**1.16**	**(1.05- 1.29)**	**3.45E-03**	**1.00**	**555.18 / 582.23 / 133.58**	**1231.6 / 1615.7 / 521.71**	**1.27**	**(1.15-1.41)**	**4.86E-06**	**0.99**	**169.97 / 175.12 / 37.887**	**889.06 / 1002 / 297.78**	**1.18**	**(0.98-1.41)**	**7.74E-02**	**0.83**	**1.21**	**(1.13-1.29)**	**2.62E-08**	**2.62E-08**	**0**	**0.45**
1q24.1	*MGST3,ALDH9A1, TMC01, LOC440700*	**rs12036561**	**165,658,994**	**A**	**G**	**428.98 / 403.03 / 111.99**	**2086.5 / 2395.5 / 715.02**	**1.17**	**(1.05- 1.29)**	**2.99E-03**	**1.00**	**555.3 / 581.9 / 133.8**	**1234.8 / 1610.5 / 523.65**	**1.27**	**(1.15-1.41)**	**4.93E-06**	**0.98**	**169.27 / 175.56 / 38.13**	**884.66 / 1007.1 / 297.07**	**1.17**	**(0.98-1.41)**	**8.18E-02**	**0.83**	**1.21**	**(1.13-1.30)**	**2.36E-08**	**2.36E-08**	**0**	**0.46**
		**rs7541817**	**165,659,714**	**C**	**T**	**427.27 / 404.2 / 112.54**	**2090.7 / 2390.6 / 715.69**	**1.16**	**(1.05- 1.28)**	**4.47E-03**	**1.00**	**557.34 / 581.36 / 132.29**	**1239.9 / 1610.9 / 518.14**	**1.27**	**(1.15-1.41)**	**5.36E-06**	**0.98**	**169.35 / 175.96 / 37.653**	**888.47 / 1002.8 / 297.5**	**1.18**	**(0.98-1.41)**	**8.01E-02**	**0.82**	**1.21**	**(1.13-1.29)**	**3.98E-08**	**3.98E-08**	**0**	**0.43**
		**rs4307543**	**165,660,029**	**G**	**T**	**428.04 / 403.04 / 112.93**	**2090.6 / 2392.7 / 713.69**	**1.16**	**(1.05- 1.28)**	**4.46E-03**	**1.00**	**557.1 / 580.62 / 133.27**	**1238.6 / 1611.9 / 518.48**	**1.27**	**(1.15-1.41)**	**5.72E-06**	**0.98**	**168.94 / 175.93 / 38.098**	**887.6 / 1001.8 / 299.38**	**1.17**	**(0.98-1.40)**	**8.54E-02**	**0.82**	**1.21**	**(1.13-1.29)**	**4.46E-08**	**4.46E-08**	**0**	**0.43**
		rs7542184	165,660,041	C	A	502.47 / 353.9 / 87.63	2421.6 / 2244.7 / 530.72	1.19	(1.07- 1.32)	1.19E-03	1.00	643.58 / 522.99 / 104.43	1496.3 / 1500.6 / 372.11	1.23	(1.10-1.36)	1.86E-04	0.98	193.44 / 161.18 / 28.344	1029.7 / 935.86 / 223.23	1.17	(0.97-1.42)	9.94E-02	0.81	1.20	(1.12-1.29)	2.09E-07	2.09E-07	0	0.89
		rs72698083	165,660,794	G	C	502.42 / 355.39 / 86.186	2427.7 / 2240.3 / 529.05	1.19	(1.07- 1.32)	1.17E-03	1.00	642.99 / 523.41 / 104.6	1495.7 / 1501.1 / 372.21	1.23	(1.10-1.36)	1.85E-04	0.98	193.1 / 161.32 / 28.552	1023.8 / 937.62 / 227.46	1.18	(0.98-1.43)	8.30E-02	0.81	1.20	(1.12-1.29)	1.73E-07	1.73E-07	0	0.91
		rs1560445	15,237,682	G	A	503.83 / 367.07 / 73.075	2452.4 / 2202.4 / 542.05	1.24	(1.11- 1.38)	1.00E-04	0.95	664 / 518 / 89	1704 / 1379 / 286	1.18	(1.06-1.32)	2.34E-03	DT	180.28 / 165.56 / 37.122	1021.9 / 935.98 / 230.95	0.96	(0.79-1.18)	7.12E-01	0.74	1.18	(1.10-1.26)	8.59E-06	1.73E-02	58	0.09
		rs1835468	15,239,211	G	A	504.1 / 368.82 / 71.057	2457.8 / 2201.3 / 537.85	1.24	(1.11- 1.38)	9.40E-05	0.96	663.82 / 517.27 / 89.904	1695.9 / 1386.8 / 286.34	1.19	(1.07-1.33)	1.56E-03	1.00	183.09 / 164.88 / 34.996	1032.2 / 932.48 / 224.14	0.98	(0.80-1.20)	8.67E-01	0.74	1.18	(1.10-1.27)	3.91E-06	5.07E-03	50	0.14
		rs13002707	15,241,629	T	A	505.02 / 367.87 / 71.094	2465.3 / 2196.6 / 535.03	1.23	(1.11- 1.37)	1.15E-04	0.97	662.91 / 519.27 / 88.821	1699.7 / 1383.2 / 286.01	1.19	(1.06-1.32)	2.08E-03	1.00	184.26 / 164.06 / 34.647	1029.3 / 934.44 / 225.09	1.00	(0.82-1.22)	9.92E-01	0.75	1.18	(1.10-1.27)	4.63E-06	1.67E-03	40	0.19
2p24.3		rs7573899	15,241,637	T	C	504.82 / 368.01 / 71.154	2465.3 / 2196.6 / 535.04	1.23	(1.11- 1.37)	1.21E-04	0.97	662.91 / 519.26 / 88.828	1699.3 / 1383.4 / 286.27	1.19	(1.07-1.33)	1.96E-03	1.00	184.22 / 164.1 / 34.659	1029 / 934.58 / 225.24	1.00	(0.82-1.22)	9.92E-01	0.75	1.18	(1.10-1.27)	4.55E-06	1.59E-03	40	0.19
		rs10196061	15,244,929	C	T	504.35 / 367.43 / 72.205	2458.2 / 2204.7 / 534.04	1.23	(1.11- 1.37)	1.37E-04	0.97	663.07 / 518.31 / 89.62	1697.2 / 1386 / 285.8	1.19	(1.07-1.33)	1.80E-03	1.00	181.25 / 167.12 / 34.605	1024 / 941.47 / 223.4	0.98	(0.80-1.20)	8.71E-01	0.75	1.18	(1.10-1.27)	5.87E-06	4.44E-03	47	0.15
		rs13385654	15,252,659	T	C	496.94 / 373.42 / 73.617	2404.4 / 2227.2 / 565.32	1.25	(1.12- 1.39)	4.28E-05	0.97	636.32 / 541.66 / 93.017	1592.4 / 1460.2 / 316.38	1.19	(1.07-1.33)	1.34E-03	0.99	179.58 / 166.4 / 36.995	998.08 / 949.72 / 241.04	1.02	(0.84-1.25)	8.23E-01	0.74	1.19	(1.11-1.28)	9.25E-07	2.52E-04	33	0.22
		rs11695054	15,254,827	G	A	505.98 / 365.89 / 72.127	2461.9 / 2200.3 / 534.8	1.23	(1.11- 1.37)	1.29E-04	0.99	665.17 / 516.82 / 89.011	1704.4 / 1380.6 / 284.05	1.19	(1.07-1.33)	1.92E-03	1.00	185.98 / 164.92 / 32.061	1030.8 / 941.86 / 216.15	1.06	(0.86-1.29)	5.97E-01	0.75	1.19	(1.11-1.28)	1.76E-06	1.76E-06	0	0.42
2p12	*REG3G*	2-79243058	79,243,058	AACAG	A	0.056 / 28.229 / 915.71	0.01 / 79.416 / 5117.6	3.87	(1.99- 7.53)	6.53E-05	0.67	0.028 / 28.494 / 1242.5	0.01 / 34.69 / 3334.3	4.41	(2.00-9.71)	2.33E-04	0.57	0.005 / 5.408 / 377.59	0.157 / 34.189 / 2154.7	0.87	(0.29-2.60)	8.08E-01	0.60	3.10	(1.96-4.92)	1.48E-06	2.35E-02	69	0.04
2q37.1	*ITM2C, GPR55, LOC151484*	rs181676155	231,787,699	A	G	0.696 / 20.657 / 922.65	0.059 / 64.344 / 5132.6	7.46	(2.92-19.09)	2.75E-05	0.59	0.112 / 29.75 / 1241.1	0.024 / 42.246 / 3326.7	3.05	(1.55-6.00)	1.22E-03	0.71	0.001 / 3.337 / 379.66	0.006 / 36.605 / 2152.4	0.43	(0.14-1.31)	1.38E-01	0.58	2.67	(1.63-4.37)	9.54E-05	2.71E-01	87	0.00
5p13.3	*PDZD2*	rs10054504	32,000,483	T	C	25 / 215 / 704	63 / 973 / 4161	1.41	(1.21- 1.65)	1.29E-05	DT	26 / 311 / 934	60 / 712 / 2597	1.24	(1.07-1.44)	4.11E-03	DT	5 / 93 / 285	35 / 472 / 1682	0.98	(0.77-1.24)	8.53E-01	DT	1.25	(1.14-1.38)	5.19E-06	3.34E-02	69	0.04
		rs78793258	85,749,252	G	A	12.513 / 138.33 / 793.16	25.438 / 615.51 / 4556.1	1.43	(1.17- 1.73)	3.61E-04	0.98	14.645 / 223.12 / 1033.2	24.034 / 495.92 / 2849	1.40	(1.17-1.68)	2.31E-04	0.98	0.34 / 69.303 / 313.35	20.822 / 304.4 / 1863.8	1.13	(0.84-1.51)	4.22E-01	0.96	1.36	(1.21-1.53)	5.68E-07	5.68E-07	0	0.38
6q14.3		rs74439948	85,766,266	G	A	11.022 / 141.43 / 791.54	25.723 / 617.84 / 4553.4	1.41	(1.16- 1.72)	4.60E-04	0.99	13.54 / 224.76 / 1032.7	25.071 / 499.19 / 2844.7	1.37	(1.14-1.63)	5.95E-04	0.99	1.129 / 66.805 / 315.07	20.11 / 303.19 / 1865.7	1.13	(0.84-1.50)	4.23E-01	0.98	1.34	(1.19-1.51)	1.64E-06	1.64E-06	0	0.42
		rs7765284	85,777,822	A	C	11 / 142 / 791	25 / 618 / 4554	1.42	(1.17- 1.72)	3.73E-04	DT	13 / 225 / 1033	25 / 498 / 2846	1.36	(1.14-1.62)	6.86E-04	DT	1 / 68 / 314	21 / 302 / 1866	1.13	(0.85-1.50)	4.00E-01	DT	1.34	(1.19-1.51)	1.50E-06	1.50E-06	0	0.42
10q21.2	*ANK3*	rs149748210	62,066,205	A	ATATATATC	211.8 / 471.97 / 260.2	990.34 / 2519.8 / 1686.7	1.26	(1.12- 1.41)	1.05E-04	0.73	314.68 / 628.25 / 328.01	838.36 / 1646.8 / 883.67	1.20	(1.07-1.34)	1.93E-03	0.76	50.472 / 178.35 / 154.16	354.13 / 1055.8 / 778.93	0.82	(0.67-1.00)	5.32E-02	0.68	1.16	(1.07-1.25)	1.21E-04	4.06E-01	85	0.00
11q23.3		rs113878013	114,385,626	C	T	0 / 19.716 / 924.28	0.438 / 44.852 / 5151.7	4.67	(2.13-10.22)	1.14E-04	0.78	0.002 / 32.69 / 1238.3	0.017 / 34.213 / 3334.8	2.98	(1.58-5.60)	7.25E-04	0.78	0 / 6.675 / 376.33	0.287 / 36.682 / 2152	0.72	(0.27-1.88)	5.01E-01	0.69	2.55	(1.65-3.96)	2.74E-05	1.00E-01	78	0.01
		rs7942540	114,815,288	A	G	0.281 / 30.459 / 913.26	0.248 / 98.727 / 5098	5.13	(2.41-10.91)	2.24E-05	0.48	0.008 / 38.808 / 1232.2	0.757 / 94.398 / 3273.8	2.89	(1.51-5.53)	1.42E-03	0.60	0.002 / 7.239 / 375.76	0.059 / 37.569 / 2151.4	1.17	(0.35-3.89)	7.97E-01	0.60	3.12	(1.98-4.93)	9.89E-07	2.91E-03	53	0.12

OR = odds ratio, CI = confidence interval, IS = imputation accuracy score (info score from IMPUTEv2), DT = directly typed

[a] nearest gene(s) = genes within 50kb of the variant with the lowest meta analysis P_{fixed}

[b] alleles are given given as risk & other allele (risk allele is determined from the UK study)

[c] genotype counts are given as AA / AB / BB where A = UK risk allele, B = UK other allele
