# Supplementary material for: Common Variation at 1q24.1 (ALDH9A1) Is a Potential Risk Factor for Renal Cancer
Source: PLoS One. 2015 Mar 31;10(3):e0122589. doi: 10.1371/journal.pone.0122589 (PMC4380462; doi:10.1371/journal.pone.0122589)
Supplement: S4 Table — (PDF) [file pone.0122589.s008.pdf]

**Supplementary Table S4: significance of the interaction terms of rs3845536 with previously published risk SNPs for RCC**

| SNP | locus | $R^2$ with rs3845536[a] | $P_{interaction}$[b] |
|---|---|---|---|
| rs7579899 | 2p21 | 6.9E-06 | 0.98 |
| rs11894252 | 2p21 | 4.6E-05 | 0.94 |
| rs9679290 | 2p21 | 4.6E-04 | 0.82 |
| rs4953346 | 2p21 | 4.8E-04 | 0.80 |
| rs12617313 | 2p21 | 1.8E-04 | 0.15 |
| rs12105918 | 2q22.3 | 1.2E-04 | 0.60 |
| rs13389578 | 2q22.3 | 2.4E-06 | 0.74 |
| rs6470588 | 8q24.1 | 3.2E-04 | 0.27 |
| rs6470589 | 8q24.1 | 3.7E-04 | 0.22 |
| rs7105934 | 11q13.3 | 5.5E-05 | 0.78 |
| rs718314 | 12p11.23 | 3.6E-05 | 0.78 |
| rs1049380 | 12p11.23 | 4.8E-05 | 0.89 |
| rs4765623 | 12q24.31 | 4.7E-05 | 0.19 |

[a] estimated from the unphased UK controls

[b] P-value for the coefficient of the interaction term in a logistic regression model that includes the main effects of both SNPs as well as their interaction term
